# Fermented table olives from Cyprus: Microbiota profile of three varieties from different regions through metabarcoding sequencing

**DOI:** 10.3389/fmicb.2022.1101515

**Published:** 2023-01-17

**Authors:** Eleni Kamilari, Dimitrios A. Anagnostopoulos, Dimitrios Tsaltas

**Affiliations:** Department of Agricultural Sciences, Biotechnology and Food Science, Cyprus University of Technology, Limassol, Cyprus

**Keywords:** table olives, fermentation, microbial diversity, high-throughput sequencing, 16S rRNA gene, ITS loci, microbial distribution, microbial biogeography

## Abstract

The knowledge about the microbial diversity of different olives varieties from diverse regions in the Mediterranean basin is limited. This work aimed to determine the microbial diversity of three different fermented olive varieties, collected from different regions in Cyprus, *via* Next Generation Sequencing (NGS) analysis. Olives were spontaneously fermented for 120 days, microbial DNA was extracted from the final products, and subjected to 16S rRNA gene and ITS1 loci metabarcoding analysis for the determination of bacterial and fungal communities, respectively. Results revealed that the bacterial profile of the studied varieties was similar, while no noteworthy differences were observed in olives from different regions. The bacterial profile was dominated by the co-existence of *Lactobacillus* and *Streptococcus*, while the genera *Lactococcus* and *Salinivibrio* and the family *Leuconostocaceae* were also present in increased relative abundances. Regarding fungal communities, the analysis indicated discrimination among the different varieties, especially in Kalamata ones. The most abundant fungi were mainly the genera *Aspergillus*, *Botryosphaeria*, *Meyerozyma*, and *Zygosaccharomyces* for Cypriot olives, the genera *Botryosphaeria*, *Saccharomyces*, *Geosmithia*, and *Wickeromyces* for Kalamata variety, while the dominant fungi in the Picual variety were mainly members of the genera *Candida*, *Penicillium*, *Saccharomyces*, *Hanseniospora* and *Botryosphaeria*. Potential microbial biomarkers that distinguish the three varieties are also proposed. Moreover, interaction networks analysis identified interactions among the key taxa of the communities. Overall, the present work provides useful information and sheds light on an understudied field, such as the comparison of microbiota profiles of different varieties from several regions in Cyprus. The study enriches our knowledge and highlights the similarities and the main differences between those aspects, booming in parallel the need for further works on this frontier, in the attempt to determine potentially olives’ microbial terroir in Cyprus. Our work should be used as a benchmark for future works in this direction.

## 1. Introduction

Table olives are among the most recognizable healthy fermented fruits and vegetables, with a great socio-economic impact, worldwide. They have been cultivated along the Mediterranean basin since antiquity, being closely related to history and local traditions. Nowadays, they are recognized as “the future food” (Bonatsou et al., 2017), as well as “more than a fermented product” (Perpetuini et al., 2020). According to the International Olive Oil Council (International Olive Oil Council [IOOC], 2004) the official term for table olive is “the product that is prepared from the sound fruits of varieties of the cultivated olive trees (*Olea europaea L.*) that are chosen for their production of olives and whose volume, shape, flesh-to-stone ratio, fine flesh, taste, firmness, and ease of detachment from the stone make them particularly suitable for processing; treated to remove their bitterness and preserved by natural fermentation; or by heat treatment, with or without the addition of preservatives; packed with or without covering liquid.” Last season (2020/2021), the global production exhibited a total of ~2,800,000 tons, while the top producer is Spain, followed by Italy and, to a lesser extent, other Mediterranean countries (e.g., Greece, Algeria, Turkey, Egypt, Portugal, and Cyprus; International Olive Oil Council [IOOC], 2020).

Successful fermentation and thus, the production of high-quality table olives is strongly dependent on the prevalence; against other microorganisms; of Lactic Acid Bacteria (LAB) and/or yeasts, the role of which is of great importance for an appropriate fermentation process, that will lead to the production of well-accepted final products (Perpetuini et al., 2020). Table olive fermentation involves complex associations among the members of the microbial ecosystem and is a non-stable procedure that takes place spontaneously until nowadays (Anagnostopoulos and Tsaltas, 2022). Even though there are different types of olives production, such as the Spanish-style, the Greek style, the Californian style, and other local-based methods (Perpetuini et al., 2020), in all cases the fermentation process occurs spontaneously, led exclusively by the indigenous microbiota coming from the raw materials (olive variety, salt, water; Heperkan, 2013; Anagnostopoulos et al., 2020a) or by contaminants during the processing stage (fermenters, tanks, patios, etc.) (Lucena-Padrós and Ruiz-Barba, 2019). This means that the microbial communities from the micro-environment where olives are cultivated, as well as olive’s cultivar., have significant roles in the microbiota formation during the fermentation process and consequently in the final product qualitative characteristics formation. Therefore, a potential geographical-dependent significance that is not well-defined until today is proposed (Anagnostopoulos and Tsaltas, 2022). This hypothesis is also reinforced by some indications that support the claim of “environmental selection,” which means that different environments are constituted by specific microbial communities that are transferred and participate in the spontaneous fermentation of several foods (Martiny et al., 2006; Argyri et al., 2020). In the case of table olives, the first attempt to shed light on this aspect was recently applied by Lucena-Padrós and Ruiz-Barba (2019) who studied the potential microbial distribution in Spanish-style green olives from several regions of Seville. Since then, a limited number of scientific research related to those aspects (comparison of different varieties and/or from different regions) has been published, such as a work performed by Kazou et al. (2020) on the microbiota of natural Black Kalamata cultivar from different regions of Greece, or the study by Argyri et al. (2020) dealing with the microbial diversity of fermented olives of two varieties from different regions in the Greek territory. As it is clearly observed, the number of works applied until now is very low, making the knowledge about those aspects very limited, even though such kind of concepts deserves more attention from the scientific community (Anagnostopoulos and Tsaltas, 2022). Connecting the microbiota composition of a fermented olive variety with a specific region may potentially lead to its promotion a as Protected Designation of Origin (PDO) or Protected Geographical Indication (PGI) product and thus, increase its added value, a fact with major economic benefits for the producers (Anagnostopoulos and Tsaltas, 2022).

Therefore, the identification and characterization of the microbial diversity of fermented olives produced by different varieties and originating from different regions represent the next big challenge in the olive sector. This will potentially highlight the uniqueness of different types of table olives produced in a specific region and explain the development of different organoleptic characteristics (taste, flavor, aroma, etc.). In the last decade, the advent of the High-Throughput Sequencing (HTS) set of techniques, such as the 16S rRNA gene and/or the ITS loci metabarcoding analyses appeared in the foreground of the scientific field to cover the limitations of conventional approaches (i.e., culture-dependent techniques), making the study of foods’ microbial communities a high-throughput and easy task, providing in parallel useful information about the microorganisms that dominate in a variety of foodstuffs in every stage of production (De Filippis et al., 2017). In a few words, this set of techniques provided a revolution in the field of food microbiology, being an important tool for an in-depth and thorough study in this direction (Cocolin et al., 2018).

In the case of table olives, NGS analysis has been applied by many scientists to determine the microbiota profile during and/or at the end of olive fermentation (Rodríguez-Gómez et al., 2017; Zinno et al., 2017; Benítez-Cabello et al., 2020; Penland et al., 2020; Anagnostopoulos et al., 2020b; López-García et al., 2021; Michailidou et al., 2021; Penland et al., 2022; Tzamourani et al., 2022). However, the vast majority of these studies are focused on the microbiota succession during fermentation or the microbiota profile of one olive variety each time and/or from one region. Furthermore, according to Argyri et al. (2020), the vast majority of investigations applied, analyses mostly brines instead of fermented olives, which is the consumed product. Moreover, studies comparing the microbial composition of different olive varieties are scarce. Additionally, the knowledge about olives from Cyprus is not well-documented. Particularly, apart from the investigation of the microbiota succession during fermentation of Picual variety from Cyprus (Anagnostopoulos et al., 2020b), no other information is available in the literature.

Thus, this work aimed to shed light on the microbial diversity of three fermented olives varieties (Cypriot, Picual, Kalamata) from different regions of Cyprus, *via* a metabarcoding approach. This is one of the first investigations dealing with those aspects and thus, the findings of the present study should be used as a pillar for further studies in this direction in the near future.

## 2. Materials and methods

### 2.1. Samples collection and processing

In total, twelve (12) pooled (from several yards) olive samples; 6 Cypriot (at the green stage of maturation), 3 Kalamata (also known as Kalamon) (at the black stage of maturation), and 3 Picual (at the green stage of maturation); were collected during the 2019–2020 season from different regions in Cyprus as described in Table 1; Supplementary Figure 1. The drupes were transferred to the laboratory of Cyprus University and Technology, Lemesos, where the fermentation took place according to the directly brining method for all varieties. Prior, drupes were cleaned with sterile water to discard potential residues, placed in plastic containers of 100 L volume, filled with brine (10% w/v NaCl, 0.3% w/v citric acid), and allowed to spontaneously ferment for 120 days at room temperature (~22°C), to simulate the procedure that is applied by the olive industry in Cyprus.

**Table 1:** Variety, sample code and geographical origin of fermented table olives.

Variety	Sample code	Geographical origin	Altitude
Cypriot	OliveC1	Famagusta (Paralimni)	75 m
	OliveC2	Nicosia (Palaichori)	930 m
	OliveC3	Lemesos (Paramali)	90 m
	OliveC4	Paphos (Statos)	940 m
	OliveC5	Paphos (Polis)	20 m
	OliveC6	Larnaca (Agklisides)	135 m
Kalamata	OliveK1	Paphos (Polis)	20 m
	OliveK2	Famagusta (Deryneia)	72 m
	OliveK3	Larnaca (Dromolaksia)	35 m
Picual	OliveP1	Famagusta (Paralimni)	75 m
	OliveP2	Nicosia (Palaichori)	930 m
	OliveP3	Larnaca (Agklisides)	135 m

### 2.2. DNA extraction and quantification

DNA extraction was applied using the DNeasy® PowerFood® Microbial Kit (MoBio Laboratories Inc., Carlsbad, CA, United States) following the manufacturer’s instructions. The extracted DNA was stored at −20°C until further processing. If the concentration of the obtained DNA was lower than 5 ng/μl, or the 260/230 ratio was lower than 1.9, indicating the presence of contaminants, then the process of DNA extraction was repeated with the addition of a small concentration of 20 μl of β − mercaptoethanol, followed by incubation at 60°C for 1 h, before the use of the DNA extraction kit mentioned above. The extracted DNA was quantified fluorometrically with Qubit 4.0 fluorometer (Invitrogen, Carlsbad, CA) using a Qubit dsDNA HS Assay Kit (Invitrogen). DNA purification was checked by measuring the ratio of absorbance A260/280 nm and A260/230 nm using a spectrophotometer (NanoDrop Thermo Scientific, United States).

### 2.3. Metabarcoding analysis of 16S rRNA gene and its loci

The metabarcoding analysis of both 16S rRNA (V3-V4 region) gene and internal transcribed spacer 1 (ITS1) loci was applied as described previously by Kamilari et al. (2022). The primers used for amplification of the V3/V4 region of the 16S rRNA gene were V3 (5-TCGTCGGCAGCGTCAGATGTGTATAAGAGACAG-3) and V4 (5-GTCTCGTGGGCTCGGAGATGTGTATAAGAGACAG-3), while ITS1 loci was amplified using the primers BITS (5-NNNNNNNNCTACCTGC GGARGGATCA-3) and B58S3 (5-GAGATCCRTTGYTRAAAGTT-3). For fungal ITS1 loci amplification and sequencing, the “Fungal Metagenomic Sequencing Demonstrated Protocol” provided by Illumina was applied. The PCR reaction and conditions were applied according to Kamilari et al. (2022) using a KAPA HiFi HotStart Ready Mix (KAPA Biosystems, United States) and a PCR Thermocycler (Bio-Rad, United States), respectively. Amplicons purification, estimation of DNA quantity and quality, and amplicons normalization was conducted as described by Papademas et al. (2021). For both bacterial 16S rRNA gene and fungal ITS1 loci sequencing, amplicons were loaded on a MiSeq 600 cycle Reagent Kit v3 (Illumina, United States) (5% PhiX) and run on a MiSeq Illumina sequencing platform.

### 2.4. Bioinformatic and statistical analysis

The raw sequences’ quality processing and filtering, the rarefaction, as well as the estimation of alpha diversity indices (Shannon, Simpson, and Chao1) and Principal Coordinate Analysis (PCoA) for estimation of beta diversity, were applied using Qiime 2 version 2020.2 (Bolyen et al., 2019), following the procedure described by Kamilari et al. (2021a) and Kamilari et al. (2020). To estimate whether different varieties had statistical differences in their beta microbial diversity, nonparametric permutational analysis of variance (PERMANOVA) (Anderson and Walsh, 2013) with 999 permutations was applied. To detect differences in alpha diversity among different samples, analysis of variance (one-way ANOVA) was applied using the SPSS 20 software (StatSoft Inc., Tulsa, OK, United States). The test used was the Least Significant Difference (LSD) at a significance level of 0.05. To assign the taxonomy to the 16S rDNA sequences into OTU the q2 − feature−classifier (Bokulich et al., 2018) against the Greengenes 13_8 99% OTUs reference sequences (McDonald et al., 2012) was used, whereas, for fungal ITS loci, the UNITE fungal internal transcribed spacer (ITS) database (8.2 release) (Abarenkov et al., 2010) was applied. The sequences were filtered to remove incomplete taxonomies that failed to be identified at the genus level. Since the Greengenes 13_8 cannot identify the new nomenclature of *Lactobacillus* genus (Zheng et al., 2020), and to get a better evaluation of the results, the 20 OTUs that appeared with higher frequency in the results were identified using BLAST SF (Altschul et al., 1990). For biomarkers discovery, the LEfSe algorithm was used as described by Kamilari et al. (2021a). Taxa with a relative abundance of less than 1% per sample were considered not important to be mentioned. Finally, a correlation network indicating strong (r > 0.6 or r < −0.6) and significant (*p* < 0.01) correlations between the microbial taxa identified in olive samples was created based on Pearson and Spearman correlations and dissimilarities based on Bray Curtis and Kullback–Leibler matrices in CoNet, using Cytoscape 3.2.1, as described by Kamilari et al. (2022). Raw sequences were deposited to the National Centre for Biotechnology Information (NCBI) in Sequence Read Archive (SRA) under the BioProject PRJNA878774.

## 3. Results

### 3.1. Bacterial diversity of fermented table olives

According to high-throughput sequencing (HTS), a total of 416,264 reads were obtained (Table 2). After filtering quality, 304,919 of them (mean of 25,409) were retained. Those high-quality reads corresponded to a total of 1,372 observed OTUs (mean of 114 each) ranging from 74 to 201. Regarding alpha diversity indices (Shannon, Simpson, Chao1), no significant differences were observed neither between different varieties (Supplementary Figure 2; Supplementary Table 1) nor among different regions (data not shown).

**Table 2:** Number of raw reads, filtered reads and alpha diversity indices of different olive varieties (Cypriot, Kalamata, Picual) from different regions, as revealed by 16S rRNA high throughput sequencing.

Sample	Raw reads	Filtered reads	Observed OTUs	Shannon	Simpson	Chao1
OliveC1	74,348	58,313	114	3.01	0.77	146
OliveC2	29,110	20,541	142	3.50	0.82	149
OliveC3	30,305	23,411	99	3.22	0.81	109
OliveC4	20,476	15,277	81	2.96	0.76	83
OliveC5	72,504	48,419	201	4.11	0.88	239
OliveC6	30,518	23,833	98	2.99	0.77	102
OliveK1	18,312	13,719	97	3.86	0.88	97
OliveK2	22,600	17,868	74	3.51	0.85	76
OliveK3	42,219	33,146	90	3.07	0.79	105
OliveP1	33,307	21,801	150	3.93	0.87	160
OliveP2	24,717	16,620	129	3.90	0.87	131
OliveP3	17,848	11,971	97	3.65	0.87	97

**Figure 1:**
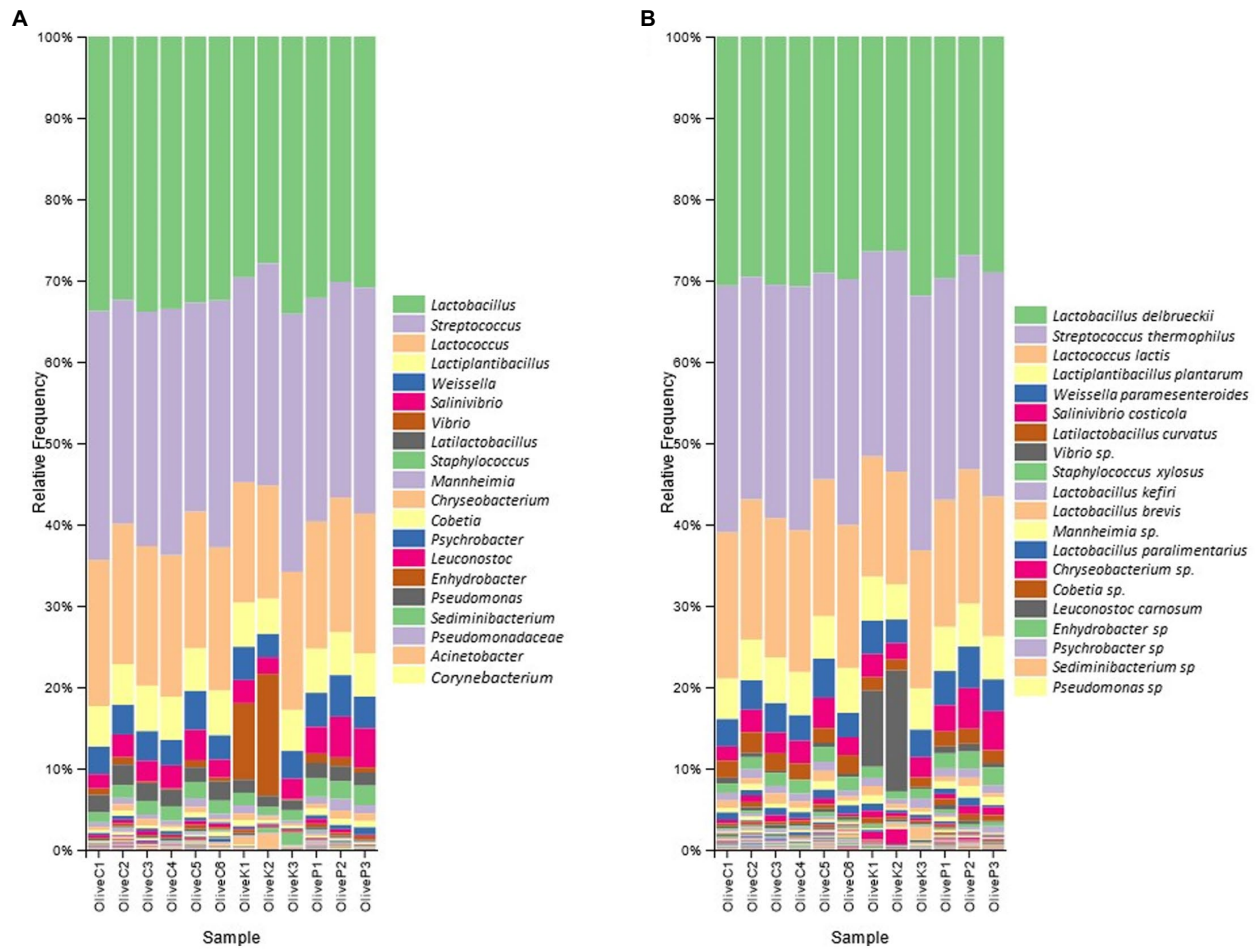
Relative abundance (%) of bacterial communities of fermented olives, Cypriot (C), Kalamata (K) and Picual (P), from different regions, as revealed by 16S rRNA high throughput sequencing. **(A)** in genus level; and **(B)** in species level.

The findings of 16S rRNA gene metataxonomic analysis are illustrated in Figure 1. In general, results indicated a quite similar bacterial profile, either between all studied varieties or among the different regions. More specifically, the dominant bacterial profile constituted by the co-existence of the genera *Lactobacillus*, and *Streptococcus*, represented mostly by the species *L. delbrueckii* and *S. thermophilus,* respectively, which amounted in summary a mean of more than 60% relative abundances, in all cases. Beyond the aforementioned, the presence of *Lactococcus* was also remarkable, reaching a mean relative abundance of about 15% in all studied samples, followed by the genera *Lactiplantibacillus, Weissella*, *Salinivibrio, Latilactobacillus* and *Staphylococcus*. To a lesser extent (relative abundance of less than 5%), the presence of *Enhydrobacter*, *Pseudomonas*, *Acinetobacter*, and *Sediminibacterium* should also be mentioned as a noteworthy part of the bacterial profile of all studied samples.

### 3.2. Fungal diversity of fermented table olives

Regarding ITS loci metabarcoding sequencing, a total of 438,227 reads were obtained (Table 3), while 228,630 high-quality reads (mean 19,052) were retained. A total of about 763 observed OTUs corresponded to the aforementioned reads, ranging from 14 to 95, with a mean value of 63 OTUs. Alpha diversity analysis indicated no significant different diversity among the studied varieties (Supplementary Figure S1; Supplementary Table S1).

**Table 3:** Number of raw reads, filtered reads, and alpha diversity indices of different olive varieties (Cypriot, Kalamata, Picual) from different regions, as revealed by ITS1 loci high throughput sequencing.

Sample	Raw reads	Filtered reads	Observed OTUs	Shannon	Simpson	Chao1
OliveC1	30,273	13,747	77	2.96	0.77	78
OliveC2	58,792	31,368	89	1.04	0.48	100
OliveC3	32,157	25,361	48	2.04	0.23	64
OliveC4	45,627	16,417	68	2.45	0.69	73
OliveC5	37,734	29,035	84	2.93	0.80	97
OliveC6	43,768	31,610	73	2.82	0.75	85
OliveK1	37,982	8,824	14	1.87	0.60	15
OliveK2	17,301	11,950	48	2.77	0.78	49
OliveK3	30,540	18,639	33	1.01	0.30	37
OliveP1	42,804	10,210	86	4.11	0.88	86
OliveP2	35,868	20,522	95	4.04	0.89	102
OliveP3	25,381	10,947	48	1.83	0.48	48

**Figure 2:**
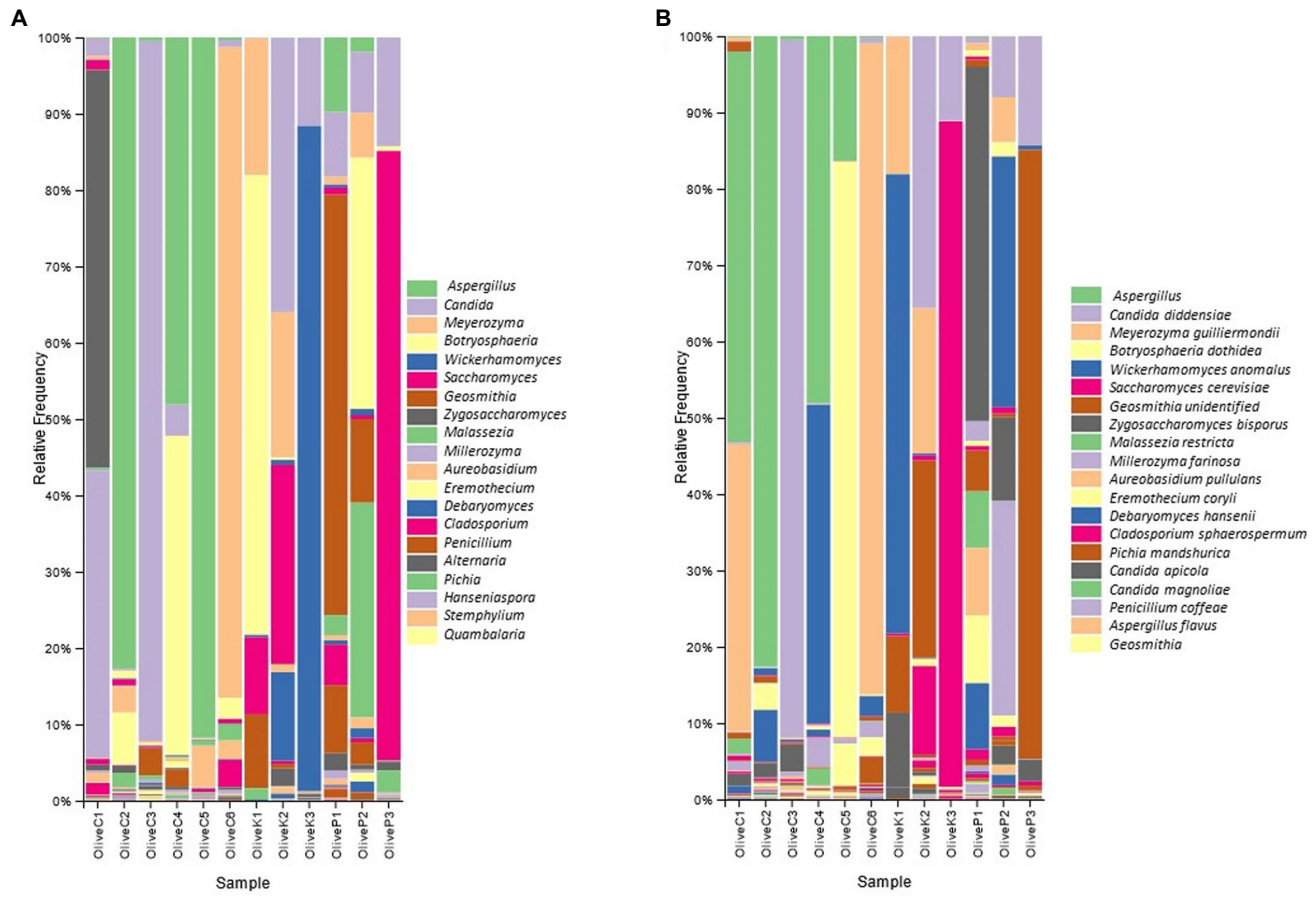
Relative abundance (%) of fungal communities of fermented olives, Cypriot (C), Kalamata (K) and Picual (P), from different regions, as revealed by ITS1 loci high throughput sequencing **(A)** in genus level; and **(B)** in species level.

According to metataxonomic analysis (Figure 2), significant differences were recorded in the fungal profile between the different varieties, even though a regional-dependent profile was also observed in some of the studied samples. More deeply, the fungal profile of Cypriot olives originating from Nicosia and Paphos was constituted mainly by the genus *Aspergillus*, exhibiting more than 50% relative abundances, while *Botryosphaeria* recorded remarkable levels, as well. On the other hand, the biota of Cypriot olives from Famagusta and Larnaca provinces indicated different profiles, since *Meyerozuma* was the dominant genus in the latter region (Larnaca), while in the former (Famagusta), this genus co-existed with *Zygosaccharomyces*, accounting, in summary, more than 90% of relative abundance. Additionally, Cypriot olives from Lemesos province recorded different fungal profiles. They constituted mainly by the genus *Candida* (~90% relative abundance), while the presence of other fungi, such as *Geosmithia* and *Aureobasidium* was more limited.

A similar observation was also recorded for the Kalamata variety since the fungal biota profile seemed to strongly be influenced by olives’ origin. More specifically, Kalamata table olives from the Paphos region were dominated by the genus *Botryosphaeria*, followed by *Meyerozyma*, while the presence of both *Saccharomyces* and *Geosmithia* is also detected. Oppositely, the biota profile of the Kalamata variety from the Famagusta region included the co-existence of the genera *Candida*, *Meyerozyma*, and *Saccharomyces*, while the presence of *Debaryomyces* was also observed. Finally, the dominance of the genus *Wickerhamomyces* is undisputed in samples collected from Larnaca province (~85% relative abundance), even though the levels of Candida should also be highlighted.

As for Picual variety, *Geosmithia* was by far the most abundant genus in samples originating from the Famagusta region, followed by the genera *Candida*, *Penicillium,* and to a lesser extent *Saccharomyces*. The latter was the most abundant microorganism in samples collected from Larnaca province, while at a lesser but noteworthy level the presence of *Candida* should also be noted. Finally, the fungal repertoire of olives collected from Nicosia was constituted by the co-existence of *Hanseniaspora* and *Botryosphaeria*, while the relative abundances of both *Candida* and *Geosmithia* are also remarkable.

### 3.3. Determination of beta diversity

**Figure 3:**
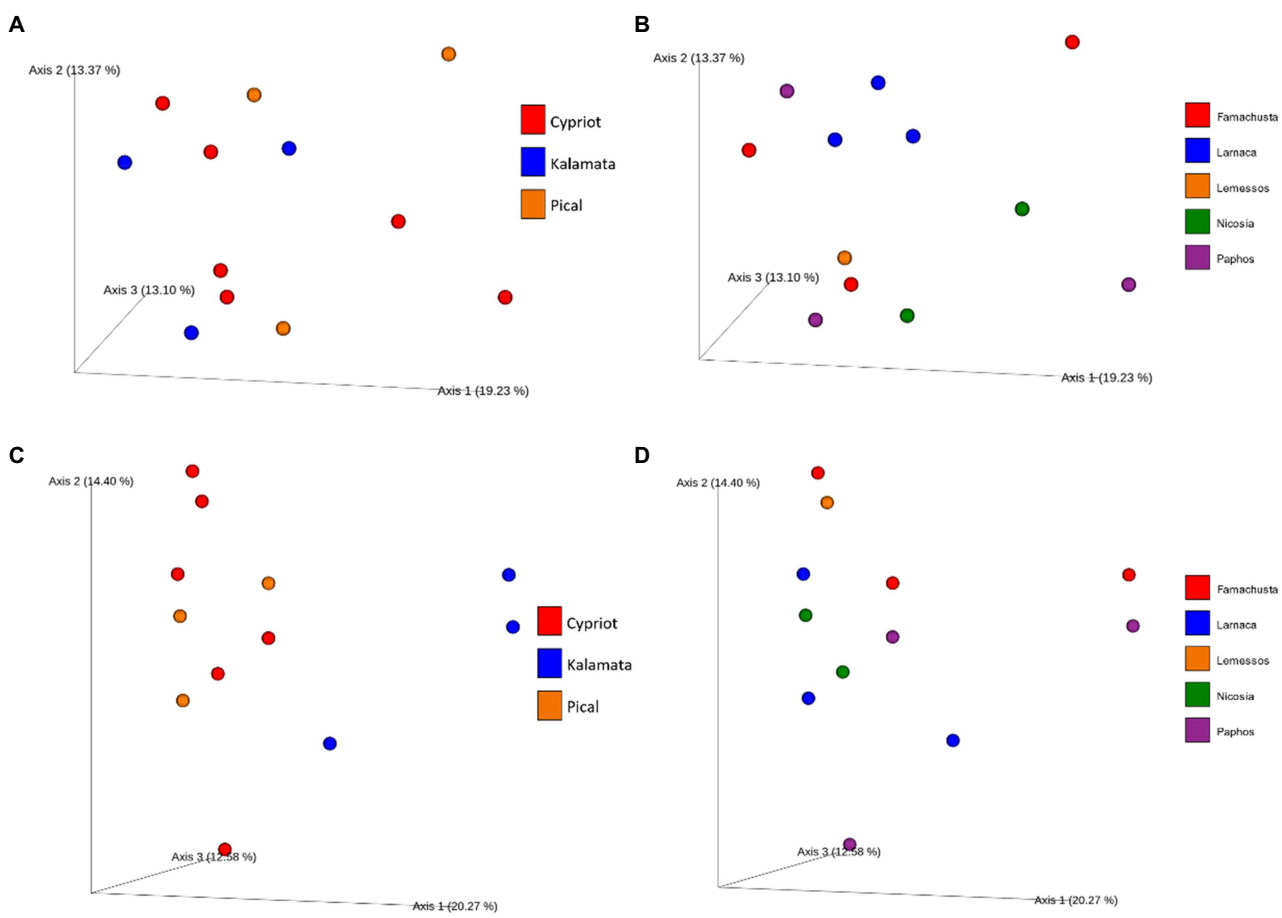
Principal coordinate analysis (PCoA) plot based on unweighted UniFrac distance. Different color corresponds to different varieties **(A)** or different regions **(B)** regarding bacterial and different varieties **(C)** or different regions **(D)** regarding fungal beta diversity.

A PCoA plot was applied to detect potential discriminations between the studied samples based on their bacterial diversity (Figures 3A,B). Results indicated that based on the bacterial diversity, no noteworthy discrimination was observed neither between the different varieties nor among the different districts. The first three components explained a total of 41.02% of the total variance (vectors 1, 2, and 3 explained 18.94, 11.14, and 10.94%, respectively).

On the other hand, PCoA of fungal biota indicated a clear distinction between samples based on variety, using the weighted unifrac distance (Figures 3C,D). More deeply, the analysis revealed clear discrimination of Kalamata from the other varieties (PERMANOVA test indicated a significant difference between Kalamata and Cypriot variety, *p* < 0.05, Supplementary Table 2). However, Cypriot and Picual varieties were not discriminated. Furthermore, it is crucial to mention that no significant differences in the fungal beta diversity were observed (*p* > 0.05) based on the Unweighted Unifrac metric among the samples from different regions. As shown in Figure 3D, samples are scattered without any noticeable separation that could indicate some significance of the region that were collected. The first three components explained a total of 47.25% of the total variance (vectors 1, 2, and 3 explained 20.27, 14.4, and 12.58%, respectively).

### 3.4. Detection of potential biomarkers

To evaluate whether the values of the identified relative abundances of microbial taxa were differentially distributed among the different olive varieties, the LEfSe algorithm was applied to both bacterial and fungal metabarcoding sequencing data.

**Figure 4:**
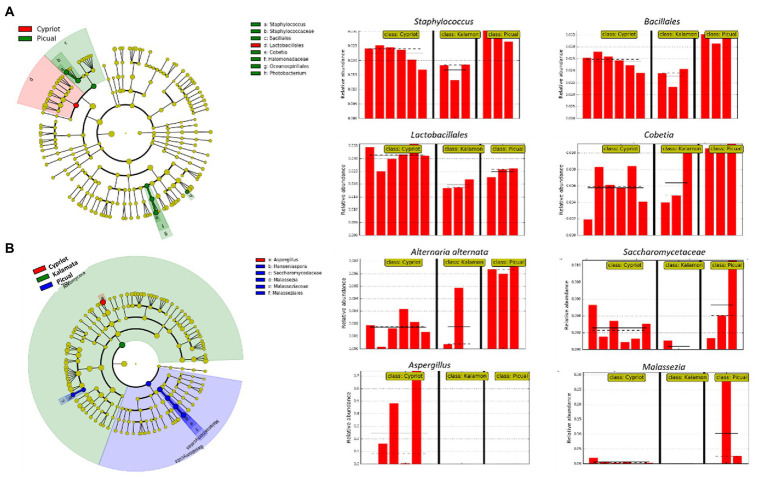
Biomarkers discovery using the LEfSe algorithm showing bacterial **(A)** and fungal **(B)** taxa that indicated significant over-representation among the different olive varieties (Cypriot, Kalamata and Picual), based on a non-parametric factorial Kruskal–Wallis (KW) sum–rank test, an (unpaired) Wilcoxon rank–sum test, and LDA. The left part of the picture depicts phylogenetic trees that map the taxonomic differences of the detected biomarkers from class (outside part of the circle) to species level (inside part of the circle), combined with a list of taxa with significantly increased representation in Cypriot olives (red color) and Picual variety (green colour regarding bacteria, blue colour regarding fungi). The right part of the picture shows histograms of the taxa that indicated significantly increased relative representation in the studied varieties, their relative abundances, and in how many samples they were detected.

Based on 16S rRNA metabarcoding, the LEfSe algorithm indicated that all studied varieties shared a similar bacterial composition, with some minor exceptions related to the representativeness percentage of particular bacteria (Figure 4A). For example, *Staphylococcus* spp. and *Cobetia* spp. exhibited a significant over-representation in the Picual cultivar compared to the other two varieties, while bacteria belonging to the order of *Lactobacillales* were more abundant in the Cypriot variety.

The LEfSE analysis of the ITS loci revealed that Cypriot olives had a sole representation of the genus *Aspergillus* (Figure 4B). On the other hand, Picual olives indicated a significantly more abundant representation of the species *Alternaria alternata* compared to the other varieties, even though this microorganism exhibited noteworthy representation in Cypriot olives, as well. No biomarkers were discovered for Kalamata olives.

### 3.5. Microbial network analysis

**Figure 5:**
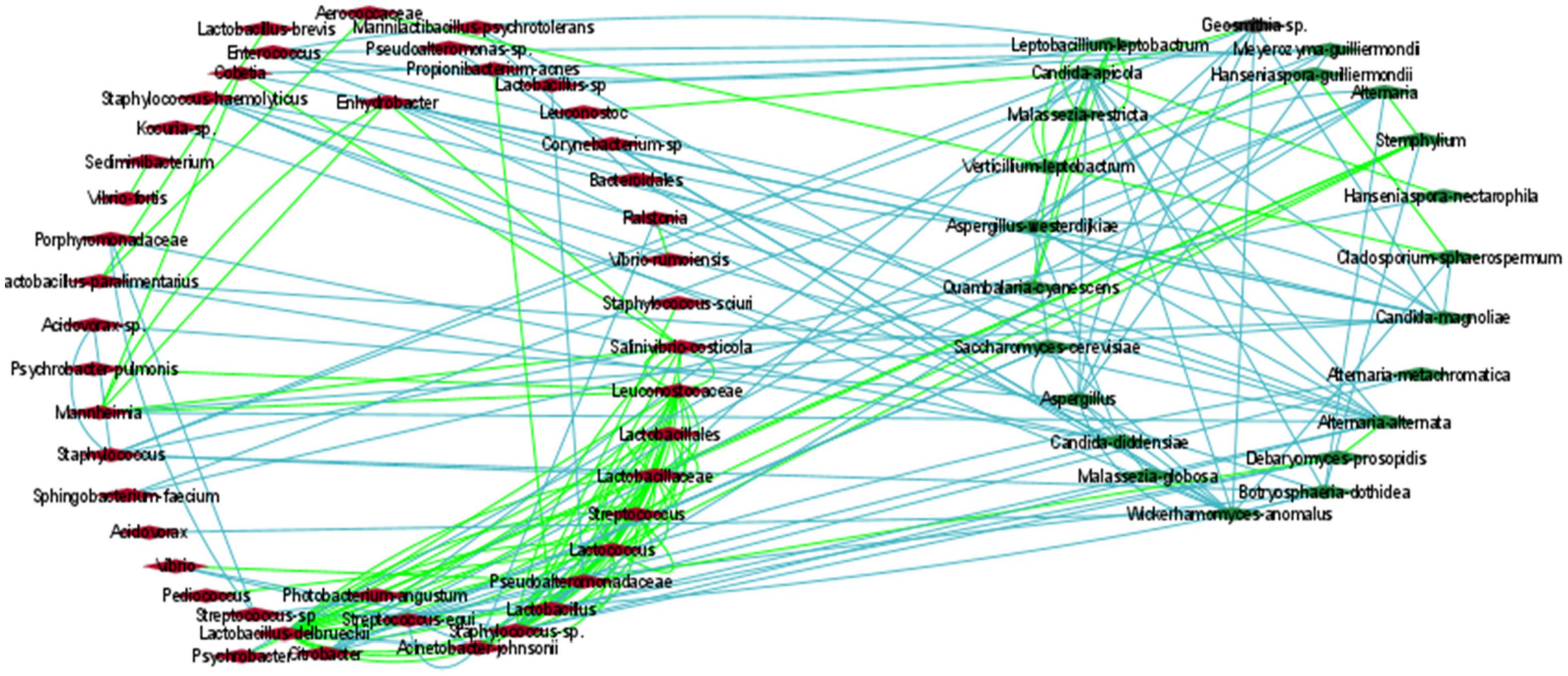
The correlation network of microbial communities in olives. Green and light-blue edges represent positive or negative correlations between two nodes, based on Spearman’s rank correlation, the Pearson correlation, and the Bray Curtis and Kullback–Leibler dissimilarity matrices. Red (left cycle) and green (right cycle) rhombus represent the bacterial and the fungal taxa, respectively.

To estimate how the members of the olive communities interacted with each other, a co-occurrence network was developed based on the Spearman and Pearson correlations and the Kullback–Leibler and Bray Curtis dissimilarity matrices in Cytoscape, (Figure 5; Supplementary Table 3). Specifically, we focus on the most abundant identified bacterial and fungal species. The analysis revealed the development of positive associations among the abundant bacterial taxa, including *L. delbrueckii*, *S. thermophilus,* and *Lactococcus lactis*. These bacteria created a co-occurrence network with other members of the family *Lactobacillaceae* and with the fungi *Stemphylium*. Additionally, *S. thermophilus,* and *L. lactis* were positively associated with *Weissella paramesenteroides* and *L. lactis* with *Pediococcus*. Moreover, *L. delbrueckii* and *S. thermophilus* were negatively associated with *Acinetobacter johnsonii*.

In contrast to bacteria, the most abundant fungi, including *Aspergillus*, *C. diddensiae*, and *M. guilliermondii* created mostly negative associations among them. *Aspergillus* was also negatively associated with *Saccharomyces cerevisiae*, *Candida apicola*, *Geosmithia*, *Wickerhamomyces anomalus*, and *Acidovorax*. *C. diddensiae* created mutual exclusion with the bacteria *Lactobacillus paralimentarius, Streptococcus equi*, *Enhydrobacter*, and a member of the family *Porphyromonadaceae*, and with the fungi *Aspergillus westerdijkiae* and *Malassezia globosa*. Finally, *M. guilliermondii* was negatively associated with *Lactobacillus kefiri*, *Cobetia*, *Psychrobacter*, and *Sphingobacterium faecium*.

## 4. Discussion

Mapping the microbial diversity of different olive varieties from diverse regions is very challenging but of great importance for both scientific and industrial communities. Such kind of research represents the vehicle to enrich our knowledge about the microbiota profile of fermented table olives (ready-to-eat) and how this relates to olive’s variety and/or origin. The final aim of this concept is to fully understand the microbiota composition and provide possible explanations about the differences in the organoleptic characteristics between several fermented olives, as well as; in some cases; between the same variety from different origins. Potential detection of olive’s microbiota uniqueness based on any of the aforementioned parameters could lead to olive’s promotion as a PDO/PGI product, surfacing in parallel the concept of microbial terroir in the field (Anagnostopoulos and Tsaltas, 2022), a concept mainly attributed to grape and wine (Bokulich et al., 2014; Kamilari et al., 2021b). However, in last years this concept has been extended to other fermented foods, in an attempt to potentially connect the specific origin (e.g., specific conditions, etc.) with the specific characteristics of the final product (Lucini et al., 2020; Kamilari et al., 2022). In the field of table olives, the first investigation in this direction was applied by Lucena-Padrós and Ruiz-Barba (2019), who studied the microbial fingerprint of Spanish-style green olives from different regions of Seville province. However, this study was applied *via* a conventional approach (culture dependent method). More recently, by exploiting the development of technology and more specifically by the advent of the NGS set of techniques, the first attempts using such approaches have already been made by some researchers. For instance, the potential effect of geographical origin on the microbiota of cv. Konservolia and cv. Halkidiki olives was recently examined by Argyri et al. (2020) *via* a metabarcoding analysis. Similarly, Kazou et al. (2020), studied the microbial fingerprint in the Kalamata variety originating from different regions in Greece. Both studies indicated promising findings and triggered the need for further works dealing with the comparison of both olives from different origins, as well as different varieties.

In the present work, the potential effects of either cultivar or geographical origin on the microbiota formation of fermented table olives in Cyprus were assessed, so that (a) to shed light on those aspects in an understudied area; and (b) to stimulate the interest for further similar research in the coming years, worldwide, to achieve the characterization of several olives’ variety typicity based on their microbial composition. For this purpose, the bacterial and fungal communities of fermented table olives produced by three varieties collected from five different districts of Cyprus, with different terroir within district, were determined *via* 16S rRNA gene and ITS1 loci metabarcoding analysis. Next Generation Sequencing represents the most ideal and rational way for the characterization of the microbial communities present in a variety of foodstuffs (Cocolin et al., 2018), including table olives (Anagnostopoulos and Tsaltas, 2022), aiming to obtain a comprehensive and reliable snapshot of microbial fingerprint and how (or if) cultivar and/or origin may influence this fingerprint (Argyri et al., 2020).

Initially, we demonstrated that phylogeny-based beta-diversity, as depicted by weighted UniFrac PCoA and confirmed by the PERMANOVA test, was able to separate the fungal diversity between Kalamata variety from the Cypriot, while no significant discrimination was achieved to neither bacterial-based diversity nor the different regions of olives. This is strong evidence that olive variety may play a crucial role, rather than olive’s origin, in the microbiota formation of fermented olives. This observation is in accordance with the literature, where for example according to Argyri et al. (2020) significant differences were observed depending on the cultivar. However, in the same study, the authors highlighted clear discrimination among the different geographical regions, as well, a fact that is not in line with the present work. This could be attributed to the small area of the country and the almost similar climate characteristics throughout the island, which may betray a homogeneity in the microbial diversity/distribution all around the areas. Anyhow, this observation is of great interest and undoubtedly deserves high attention in the near future. Furthermore, bacterial microbiota was quite similar among all studied variables from all regions and thus, the discrimination was impossible. This was also confirmed using the LEfSE algorithm. On the other hand, our study indicates that yeast microbiota could be a more reliable “biomarker tool” to study and discriminate different olives’ varieties. This is in line with a previous work by Kazou et al. (2020), since the authors noted that satisfactory discrimination could be obtained by fungal ITS metataxonomic analysis. LEfSE algorithm revealed that some fungal microorganisms such as *Aspergillus* and *Alternaria alternata*, exhibited an over-representation of one of the studied varieties (e.g., *Alternaria alternata* was over-represented in Picual olives), or in some cases, a microorganism was solely detected in one of the varieties (e.g., *Aspergillus* was solely identified in Cypriot olives). Interestingly, network association analysis revealed that *Aspergillus* sp., *C. diddensiae*, and *M. guilliermondii* (the most abundant identified fungal species) created negative associations among them. Even if more work is needed to confirm these indications, these findings open up new horizons for further attention and deeper investigation into those aspects. Using some biomarkers (in this case fungal), and further microbial diversity network association analysis, it would be possible to ensure both the distinction of the different olives’ varieties, as well as their authenticity, thus increasing its added value in global trade. In this sense, it is clear that thorough research in this direction is of great importance in the next few years.

The results of 16S rRNA metabarcoding analysis revealed an undisputed co-dominance of the genera *Lactobacillus* and *Streptococcus*, while *Lactococcus*, as well as bacteria belonging to the family of *Leuconostocaceae* also exhibited noteworthy abundances. Network association analysis indicated that these taxa were positively associated with each other. The importance of several species belonging to the genus *Lactiplantibacillus* (former-*Lactobacillus*) and their major role as key players in olives’ fermentation has been extensively noted by many works on both green and black olives, using both conventional (culture-dependent) and modern (NGS) approaches (Doulgeraki et al., 2013; Bleve et al., 2014; Lucena-Padrós et al., 2014; Bleve et al., 2015; De Angelis et al., 2015; Randazzo et al., 2017; Benítez-Cabello et al., 2020; Anagnostopoulos et al., 2020b; López-García et al., 2021; Correa-Galeote et al., 2022; López-García et al., 2022). It is crucial to mention that several *Lactiplantibacillus* and *Lactobacillus* species produce bacteriocins, eliminating the development and thus the presence of spoilages and pathogens in the final product (Doulgeraki et al., 2013). Indeed, the network association analysis revealed their negative interaction with the spoilage *Acinetobacter johnsonii*. Furthermore, they exhibit remarkable essential biotechnological characteristics for the improvement of the fermentation process and enrich the organoleptic characteristics of the final product (De Angelis et al., 2015), while their probiotic potential should also be highlighted (Bautista-Gallego et al., 2013; Montoro et al., 2016; Guantario et al., 2018). Regarding *Streptococcus*, the role of this LAB genus is more limited in olives’ fermentation, even though it has been repeatably noted as the main part of table olives’ microbiota (Randazzo et al., 2012; Heperkan, 2013). The genus *Lactococcus* has also a limited role since it is rarely found, or found at low abundances, during or at the end of olive’s fermentation, as for example detected by De Angelis et al. (2015) in Bella di Cerignola table olives, or by Zinno et al. (2017) in Nocellara del Belice variety or by Medina et al. (2018) in Aloreña de Málaga table olive. However, the relatively high abundance of this genus found herein should gain more attention, since several *Lactococcus* species, at least from dairy products, have been previously characterized as very promising probiotics (Lee et al., 2015; Yerlikaya, 2019), and thus, their high presence in fermented table olives should be desirable. Finally, regarding several heterofermentative cocci belonging to the family of Leuconostocaceae, even though this bacterial group (especially *Leuconostoc* spp.) has been characterized as low acid-producing microorganisms, thus not contributing strongly to the fermentation process, its presence is very usual in several olives from different regions (De Bellis et al., 2010; Hurtado et al., 2012). However, it is crucial to mention that its probiotic potential has been previously noted by Botta et al. (2014), indicating an important role of such bacteria, considering olives’ functional properties to human health.

Moreover, the findings of ITS1 loci metabarcoding analysis indicated the presence of several fungal taxa that are usually found in several table olives, worldwide. However, it is crucial to mention that some of those microorganisms were well-shared (e.g., *Botryosphaeria*, *Candida*, *Geosmithia*, *Saccharomyces*, etc.) among the studied varieties, while others, such as *Aspergillus*, were solely detected in one of the studied varieties. The latter indicates that olive variety shape somehow the microbial composition of the final product and as a consequence, contributes to some extent to the formation of the sensorial attributes of table olive. It should also be mentioned that despite the fact that the role of fungal microorganisms in olive fermentation has been revised in the last two decades (Arroyo-López et al., 2012; Anagnostopoulos et al., 2017) and nowadays their presence in fermented olives is considered desirable (mainly due to their crucial enzymatic capacity, as well as their probiotic potential), herein we detected some genera, the presence of which should be taken into consideration in future works. For example, beyond the presence of desirable yeast taxa (i.e., *Candida*, *Saccharomyces*, etc.) that have proven contributor effects to both olives’ sensorial formation, as well as to the insurance of an appropriate fermentation process, the presence of both *Botryosphaeria* and *Geosmithia* indicates a potential fungal distribution throughout the island that may correspond to olive tree disease. The former fungus is closely related to the so-called Dalmatian disease of the olive tree and is usually found throughout the Mediterranean basin (Moral et al., 2017), while the latter fungus is associated with many insect species that invade the phloem or sapwood of various plants/trees (Kolařík et al., 2007). Even if the role of both microorganisms during olive fermentation as well as in the final product is not well-documented, the present work constitutes the tip of the spear for further research on this aspect.

Overall, the present work provides useful information about the microbial composition of three olive varieties originating from different regions all around Cyprus, a very understudied area that deserves much more attention in the coming years. In future perspectives, more samples and/or olive varieties originating from different areas of the Mediterranean basin should be examined and compared *via* metabarcoding approaches, so that (a) to be delineated the microbial borders between the different cultivars and (b) to stand out several microbial terroirs based on table olives microbiota. Potential detection of microbial terroir could correspond to olives’ unique sensorial attributes (e.g., flavor, aroma, color, taste, etc.) and this could lead to their promotion as PDO or PGI products, with invaluable benefits for the producers. Further, the implementation of other meta-omics (metatranscriptomics, metaproteomics, metabolomics, etc.) approaches and the combination of them (multi-omics) will greatly contribute to this attempt and as a consequence, future studies should be focused on this direction.

## 5. Conclusion

The present work provides a first glance and sheds light on the microbiota profile of different olive varieties from different regions in Cyprus. Metabarcoding analysis was very useful to obtain a holistic snapshot of both bacterial and fungal communities. Even though the discrimination of olives microbiota from different origins was not achieved, the present work revealed a complex and different fungal profile between the studied varieties, while some key taxa associated with specific cultivars were also highlighted. The present study should stimulate the scientific interest for more similar works, while our findings should be used as a benchmark for further investigation in an attempt to highlight key microbial taxa that are associated with a specific cultivar and/or region. The implementation of multi-omics will strengthen the impact of such investigation to highlight the typicity of different table olives based on their microbial composition.

## Data availability statement

The datasets presented in this study can be found in online repositories. The names of the repository/repositories and accession number(s) can be found in the article/Supplementary material.

## Author contributions

DT: conceptualization. EK and DA: methodology. EK: formal analysis, investigation, and data curation. DA: writing–original draft preparation. DT: resources, supervision, project administration, and funding acquisition. DT and EK: writing–review, and editing. All authors have read and agreed to the published version of the manuscript.

## Funding

This study received funding from INTERREG Greece – Cyprus 2014–2020 Program, Project AGRO-ID, which is co-funded by the European Union (ERDF) and National Resources of Greece and Cyprus.

## Conflict of interest

The authors declare that the research was conducted in the absence of any commercial or financial relationships that could be construed as a potential conflict of interest.

## Publisher’s note

All claims expressed in this article are solely those of the authors and do not necessarily represent those of their affiliated organizations, or those of the publisher, the editors and the reviewers. Any product that may be evaluated in this article, or claim that may be made by its manufacturer, is not guaranteed or endorsed by the publisher.
