# Supplementary material for: Fermented table olives from Cyprus: Microbiota profile of three varieties from different regions through metabarcoding sequencing
Source: Front Microbiol. 2023 Jan 17;13:1101515. doi: 10.3389/fmicb.2022.1101515 (PMC9886855; doi:10.3389/fmicb.2022.1101515)
Supplement: Supplementary file 1 [file Data_Sheet_1.PDF]

Supplementary Material

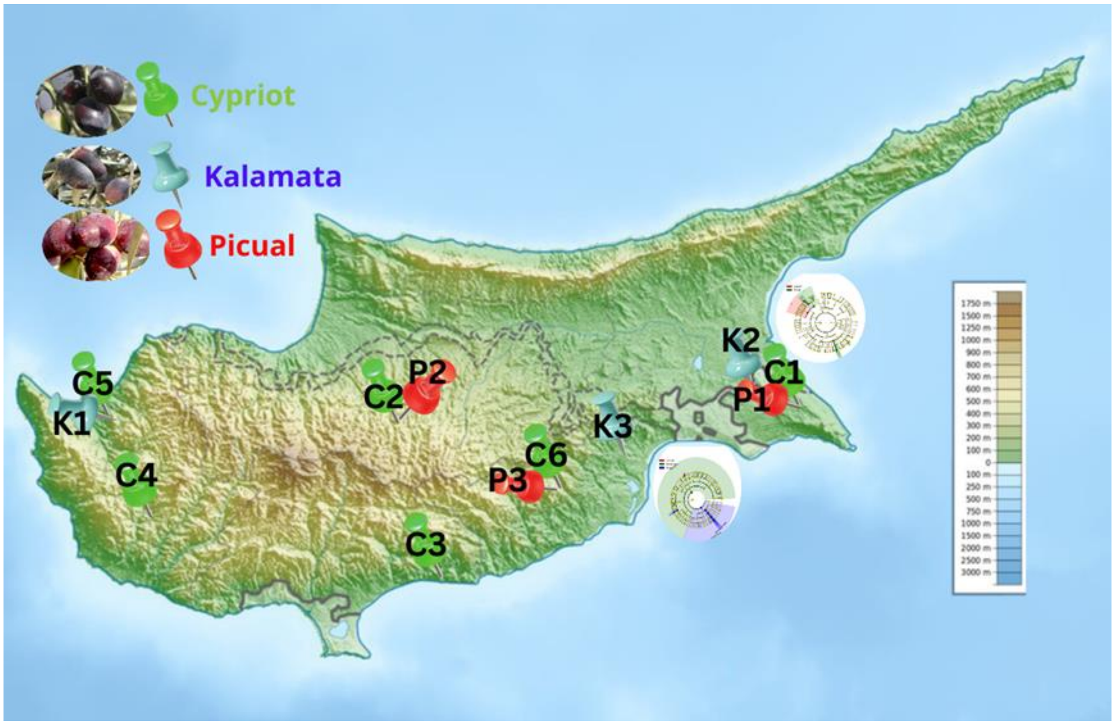

Supplementary Figure 1. Map of Cyprus with olives sampling sites.

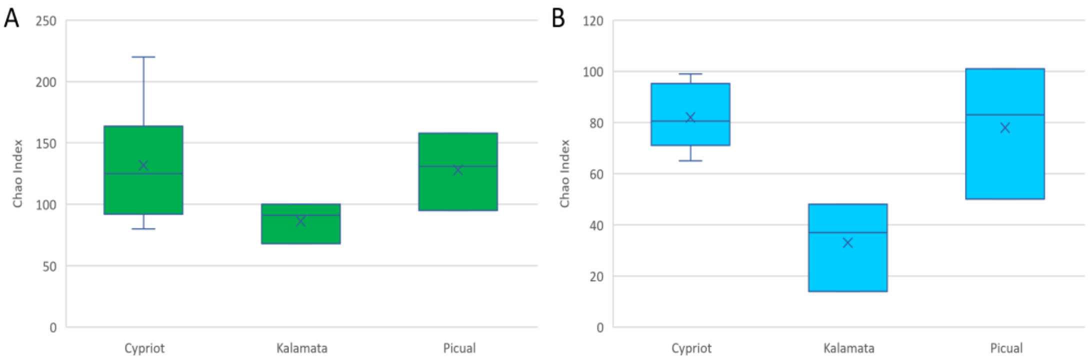

Supplementary Figure 2. Comparison of (A) bacterial and (B) fungal alpha diversities of olive varieties, based on the Chao1 index. Statistical analysis was performed using the Kruskal–Wallis test.

Supplementary Table 1. Comparison of the alpha microbial diversity of olive varieties based on the Kruskal–Wallis test.

Microorganism	Alpha diversity Index	Group 1	Group 2	H	p-value	q-value
**Bacteria**	Shannon	Cypriot (n=6)	Kalamata (n=3)	0.067	0.796	0.796
		Cypriot (n=6)	Picual (n=3)	2.400	0.121	0.190
		Kalamata (n=3)	Picual (n=3)	2.333	0.127	0.190
	Chao	Cypriot (n=6)	Kalamata (n=3)	2.400	0.121	0.190
		Cypriot (n=6)	Picual (n=3)	0.000	1.000	1.000
		Kalamata (n=3)	Picual (n=3)	2.333	0.127	0.190
	Simpson	Cypriot (n=6)	Kalamata (n=3)	0.267	0.606	0.606
		Cypriot (n=6)	Picual (n=3)	2.400	0.121	0.182
		Kalamata (n=3)	Picual (n=3)	3.857	0.050	0.149
**Fungi**	Shannon	Cypriot (n=6)	Kalamata (n=3)	1.067	0.302	0.302

		Cypriot (n=6)	Picual (n=3)	1.067	0.302	0.302
		Kalamata (n=3)	Picual (n=3)	1.190	0.275	0.302
	Chao	Cypriot (n=6)	Kalamata (n=3)	5.400	0.020	0.060
		Cypriot (n=6)	Picual (n=3)	0.000	1.000	1.000
		Kalamata (n=3)	Picual (n=3)	3.857	0.050	0.074
	Simpson	Cypriot (n=6)	Kalamata (n=3)	0.067	0.796	0.796
		Cypriot (n=6)	Picual (n=3)	1.667	0.197	0.413
		Kalamata (n=3)	Picual (n=3)	1.190	0.275	0.413

Supplementary Table 2. Comparison of olive varieties microbial beta diversity, based on the permanova-pairwise test.

Microorganism	Unifrac distance	Group 1	Group 2	Sample size	Permutations	pseudo-F	p-value	q-value
**Bacteria**	Unweighted	Cypriot	Kalamata	9	999	0.847	0.772	0.772
		Cypriot	Picual	9	999	1.027	0.381	0.720

		Kalamata	Picual	6	999	0.995	0.480	0.720
	Weighted	Cypriot	Kalamata	9	999	1.065	0.377	0.377
		Cypriot	Picual	9	999	7.643	0.047	0.141
		Kalamata	Picual	6	999	9.943	0.098	0.147
**Fungi**	Unweighted	Cypriot	Kalamata	9	999	2.320	0.014	0.042
		Cypriot	Picual	9	999	1.206	0.155	0.155
		Kalamata	Picual	6	999	1.760	0.111	0.155
	Weighted	Cypriot	Kalamata	9	999	1.622	0.214	0.321
		Cypriot	Picual	9	999	0.961	0.324	0.324
		Kalamata	Picual	6	999	1.703	0.177	0.321

Supplementary Table 3. Identified interactions between microbes in olives in the co-occurrence network.

**Label**	**interactionType**	**cooc_method**	**weight**

**Salinivibrio-costicola->Leuconostocaceae**	copresence	dist_kullbackleibler	0.050355
**Lactobacillales->Lactobacillaceae**	copresence	dist_kullbackleibler	0.03437
**Streptococcus->Lactococcus**	copresence	correl_pearson	0.972658
**Pseudoalteromonadaceae->Lactobacillus**	mutualExclusion	correl_pearson	-0.51583
**Staphylococcus-sp.->Lactobacillus**	copresence	dist_kullbackleibler	0.055168
**Acinetobacter-johnsonii->Streptococcus-equi**	mutualExclusion	correl_pearson	-0.53967
**Cladosporium-sphaerospermum->Candida-magnoliae**	mutualExclusion	dist_kullbackleibler	16.72899
**Lactobacillales->Streptococcus**	copresence	dist_bray	0.068584
**Citrobacter->Alternaria-metachromatica**	mutualExclusion	correl_pearson	-0.6152
**Alternaria-alternata->Debaryomyces-prosopidis**	copresence	correl_pearson	0.899286
**Photobacterium-angustum->Psychrobacter**	copresence	correl_spearman	0.841246
**Lactobacillus-delbrueckii->Leuconostocaceae**	copresence	dist_kullbackleibler	0.037755
**Botryosphaeria-dothidea->Streptococcus-sp**	mutualExclusion	correl_pearson	-0.65591
**Wickerhamomyces-anomalus->Malassezia-globosa**	mutualExclusion	dist_bray	0.991515

**Lactococcus->Pediococcus**	copresence	correl_pearson	0.871929
**Vibrio->Staphylococcus-sp.**	mutualExclusion	correl_pearson	-0.62494
**Leuconostocaceae->Lactobacillus**	copresence	dist_bray	0.061724
**Candida-diddensiae->Aspergillus**	mutualExclusion	dist_bray	0.991871
**Lactobacillales->Lactobacillus-delbrueckii**	copresence	dist_bray	0.069703
**Saccharomyces-cerevisiae->Wickerhamomyces-anomalus**	mutualExclusion	dist_kullbackleibler	23.80793
**Acidovorax->Wickerhamomyces-anomalus**	mutualExclusion	dist_bray	0.991537
**Quambalaria-cyanescens->Sphingobacterium-faecium**	mutualExclusion	correl_spearman	-0.62351
**Lactococcus->Lactobacillales**	copresence	dist_kullbackleibler	0.02777
**Saccharomyces-cerevisiae->Aspergillus**	mutualExclusion	dist_bray	0.985445
**Staphylococcus->Botryosphaeria-dothidea**	mutualExclusion	dist_bray	0.988502
**Aspergillus-westerdijkiae->Candida-magnoliae**	mutualExclusion	dist_kullbackleibler	23.67016
**Lactobacillaceae->Lactococcus**	copresence	dist_bray	0.028805
**Verticillium-leptobactrum->Malassezia-restricta**	copresence	correl_pearson	0.985776

**Alternaria-alternata->Candida-apicola**	mutualExclusion	dist_kullbackleibler	17.62483
**Leptobacillium-leptobactrum->Verticillium-leptobactrum**	copresence	correl_spearman	0.921786
**Malassezia-restricta->Leptobacillium-leptobactrum**	copresence	correl_spearman	0.834386
**Citrobacter->Alternaria-metachromatica**	mutualExclusion	correl_spearman	-0.62384
**Aspergillus->Wickerhamomyces-anomalus**	mutualExclusion	dist_bray	0.992287
**Staphylococcus-sp.->Lactococcus**	copresence	dist_bray	0.095178
**Saccharomyces-cerevisiae->Aspergillus**	mutualExclusion	dist_kullbackleibler	17.07245
**Lactobacillaceae->Lactobacillus**	copresence	dist_kullbackleibler	0.02505
**Aspergillus-westerdijkiae->Candida-magnoliae**	mutualExclusion	dist_bray	0.997559
**Alternaria-alternata->Mannheimia**	mutualExclusion	correl_pearson	-0.5726
**Alternaria-alternata->Staphylococcus-sp.**	mutualExclusion	correl_pearson	-0.62656
**Lactococcus->Lactobacillus**	copresence	dist_bray	0.06733
**Lactobacillaceae->Lactobacillus-delbrueckii**	copresence	correl_pearson	0.956153
**Lactobacillales->Lactobacillus-delbrueckii**	copresence	dist_kullbackleibler	0.036242
**Aspergillus->Wickerhamomyces-anomalus**	mutualExclusion	dist_kullbackleibler	19.90246

**Leuconostocaceae->Psychrobacter-pulmonis**	copresence	correl_spearman	0.865578
**Acidovorax-sp.->Aspergillus**	mutualExclusion	dist_bray	0.995614
**Geosmithia-sp.->Candida-apicola**	copresence	correl_pearson	0.864724
**Acidovorax-sp.->Staphylococcus**	mutualExclusion	dist_bray	1
**Lactobacillus-paralimentarius->Candida-diddensiae**	mutualExclusion	correl_spearman	-0.60839
**Streptococcus->Acinetobacter-johnsonii**	mutualExclusion	correl_spearman	-0.66809
**Porphyromonadaceae->Candida-diddensiae**	mutualExclusion	correl_spearman	-0.65689
**Lactococcus->Lactobacillales**	copresence	correl_pearson	0.884761
**Vibrio->Pseudoalteromonadaceae**	copresence	correl_pearson	0.989962
**Vibrio-fortis->Sediminibacterium**	copresence	correl_spearman	0.839991
**Malassezia-globosa->Candida-diddensiae**	mutualExclusion	dist_bray	0.988601
**Geosmithia-sp.->Staphylococcus**	mutualExclusion	dist_kullbackleibler	17.68017
**Lactobacillus-paralimentarius->Saccharomyces-cerevisiae**	mutualExclusion	correl_pearson	-0.54616
**Lactobacillaceae->Leuconostocaceae**	copresence	dist_bray	0.058626

**Kocuria-sp.->Botryosphaeria-dothidea**	mutualExclusion	correl_pearson	-0.52946
**Lactococcus->Lactobacillus-delbrueckii**	copresence	correl_spearman	0.839161
**Geosmithia-sp.->Wickerhamomyces-anomalus**	mutualExclusion	dist_kullbackleibler	17.77693
**Salinivibrio-costicola->Leuconostocaceae**	copresence	dist_bray	0.08144
**Wickerhamomyces-anomalus->Staphylococcus-haemolyticus**	mutualExclusion	dist_bray	0.989979
**Aspergillus-westerdijkiae->Geosmithia-sp.**	mutualExclusion	dist_kullbackleibler	17.34133
**Lactococcus->Leuconostocaceae**	copresence	dist_kullbackleibler	0.034925
**Lactococcus->Lactobacillus-delbrueckii**	copresence	correl_pearson	0.984081
**Streptococcus->Lactobacillus-delbrueckii**	copresence	correl_spearman	0.951049
**Alternaria-alternata->Candida-apicola**	mutualExclusion	dist_bray	0.986791
**Staphylococcus-sp.->Lactobacillaceae**	copresence	dist_bray	0.083647
**Lactococcus->Lactobacillales**	copresence	dist_bray	0.059664
**Meyerozyma-guilliermondii->Cobetia**	mutualExclusion	correl_pearson	-0.51672
**Staphylococcus-sp.->Debaryomyces-prosopidis**	mutualExclusion	correl_pearson	-0.53862
**Saccharomyces-cerevisiae->Citrobacter**	mutualExclusion	correl_spearman	-0.68622

**Verticillium-leptobactrum->Hanseniaspora-guilliermondii**	copresence	correl_spearman	0.832472
**Aspergillus-westerdijkiae->Candida-magnoliae**	mutualExclusion	correl_spearman	-0.64662
**Citrobacter->Alternaria**	mutualExclusion	correl_pearson	-0.5844
**Aspergillus-westerdijkiae->Saccharomyces-cerevisiae**	mutualExclusion	dist_kullbackleibler	19.87518
**Candida-apicola->Aspergillus**	mutualExclusion	dist_bray	0.987333
**Pseudoalteromonadaceae->Streptococcus-equi**	mutualExclusion	correl_pearson	-0.51385
**Aspergillus-westerdijkiae->Enterococcus**	mutualExclusion	dist_kullbackleibler	18.17511
**Lactobacillaceae->Lactobacillus**	copresence	dist_bray	0.057465
**Acidovorax-sp.->Staphylococcus**	mutualExclusion	correl_spearman	-0.65217
**Lactobacillus->Streptococcus**	copresence	dist_bray	0.078165
**Leptobacillium-leptobactrum->Verticillium-leptobactrum**	copresence	correl_pearson	0.996084
**Cobetia->Candida-magnoliae**	mutualExclusion	correl_spearman	-0.6526
**Lactobacillus-brevis->Lactobacillus-paralimentarius**	copresence	dist_bray	0.089615
**Staphylococcus-haemolyticus->Alternaria-alternata**	mutualExclusion	dist_kullbackleibler	16.27714

**Staphylococcus-sp.->Lactobacillaceae**	copresence	dist_kullbackleibler	0.039875
**Aerococcaceae->Cladosporium-sphaerospermum**	copresence	correl_spearman	0.832842
**Salinivibrio-costicola->Staphylococcus-sp.**	copresence	dist_bray	0.075231
**Citrobacter->Alternaria**	mutualExclusion	correl_spearman	-0.6873
**Lactococcus->Lactobacillus-delbrueckii**	copresence	dist_kullbackleibler	0.002227
**Wickerhamomyces-anomalus->Cladosporium-sphaerospermum**	mutualExclusion	dist_kullbackleibler	16.33642
**Lactobacillales->Streptococcus**	copresence	dist_kullbackleibler	0.040354
**Enterococcus->Leptobacillium-leptobactrum**	mutualExclusion	correl_spearman	-0.65217
**Lactobacillaceae->Lactococcus**	copresence	dist_kullbackleibler	0.006044
**Meyerozyma-guilliermondii->Aspergillus**	mutualExclusion	dist_bray	0.990974
**Enterococcus->Leptobacillium-leptobactrum**	mutualExclusion	dist_bray	1
**Citrobacter->Candida-apicola**	mutualExclusion	dist_bray	0.994047
**Aerococcaceae->Lactobacillus-paralimentarius**	copresence	correl_spearman	0.826588
**Mannheimia->Leuconostocaceae**	copresence	correl_spearman	0.818182

**Lactobacillaceae->Stemphylium**	copresence	correl_pearson	0.945547
**Wickerhamomyces-anomalus->Candida-apicola**	mutualExclusion	dist_bray	0.985336
**Lactobacillaceae->Streptococcus**	copresence	dist_kullbackleibler	0.009229
**Saccharomyces-cerevisiae->Marinilactibacillus-psychrotolerans**	mutualExclusion	correl_spearman	-0.60864
**Pseudoalteromonas-sp.->Wickerhamomyces-anomalus**	mutualExclusion	dist_bray	0.989576
**Staphylococcus-sp.->Leuconostocaceae**	copresence	dist_kullbackleibler	0.032906
**Salinivibrio-costicola->Leuconostocaceae**	copresence	correl_spearman	0.846154
**Enhydrobacter->Mannheimia**	copresence	correl_spearman	0.909091
**Malassezia-restricta->Leptobacillium-leptobactrum**	copresence	dist_bray	0.090331
**Aspergillus->Geosmithia-sp.**	mutualExclusion	dist_bray	0.994031
**Streptococcus->Lactobacillus-delbrueckii**	copresence	dist_bray	0.017513
**Quambalaria-cyanescens->Malassezia-restricta**	copresence	correl_pearson	0.930954
**Aspergillus-westerdijkiae->Alternaria-alternata**	mutualExclusion	dist_kullbackleibler	21.29424

**Quambalaria-cyanescens->Leptobacillium-leptobactrum**	copresence	correl_pearson	0.904042
**Lactobacillaceae->Lactobacillus-delbrueckii**	copresence	dist_bray	0.031792
**Staphylococcus-haemolyticus->Alternaria-alternata**	mutualExclusion	dist_bray	1
**Streptococcus->Leuconostocaceae**	copresence	dist_bray	0.095408
**Streptococcus->Lactococcus**	copresence	correl_spearman	0.811189
**Candida-apicola->Staphylococcus**	mutualExclusion	dist_bray	0.988812
**Candida-magnoliae->Mannheimia**	mutualExclusion	correl_spearman	-0.77587
**Mannheimia->Salinivibrio-costicola**	copresence	correl_spearman	0.909091
**Streptococcus->Lactobacillus-delbrueckii**	copresence	dist_kullbackleibler	0.001787
**Lactobacillaceae->Streptococcus**	copresence	dist_bray	0.041362
**Vibrio->Streptococcus-equi**	mutualExclusion	correl_pearson	-0.50582
**Candida-apicola->Candida-magnoliae**	mutualExclusion	dist_kullbackleibler	17.76772
**Enhydrobacter->Salinivibrio-costicola**	copresence	correl_spearman	0.818182
**Alternaria-alternata->Enhydrobacter**	mutualExclusion	correl_pearson	-0.54397
**Botryosphaeria-dothidea->Streptococcus-sp**	mutualExclusion	correl_spearman	-0.87916

**Lactobacillus-delbrueckii->Lactobacillus**	copresence	dist_kullbackleibler	0.026666
**Candida-apicola->Hanseniaspora-nectarophila**	copresence	correl_pearson	0.904974
**Aspergillus-westerdijkiae->Enterococcus**	mutualExclusion	dist_bray	0.999138
**Stemphylium->Botryosphaeria-dothidea**	mutualExclusion	dist_kullbackleibler	16.59411
**Candida-apicola->Aspergillus-westerdijkiae**	mutualExclusion	dist_kullbackleibler	18.16138
**Lactobacillus->Propionibacterium-acnes**	copresence	correl_spearman	0.819379
**Aspergillus-westerdijkiae->Candida-diddensiae**	mutualExclusion	dist_kullbackleibler	16.92807
**Candida-magnoliae->Salinivibrio-costicola**	mutualExclusion	correl_spearman	-0.6091
**Debaryomyces-prosopidis->Lactobacillus**	mutualExclusion	correl_pearson	-0.51673
**Staphylococcus-haemolyticus->Alternaria-alternata**	mutualExclusion	correl_spearman	-0.65217
**Lactobacillales->Lactobacillaceae**	copresence	dist_bray	0.076644
**Staphylococcus-sp.->Lactococcus**	copresence	dist_kullbackleibler	0.054924
**Streptococcus->Lactobacillus-delbrueckii**	copresence	correl_pearson	0.987478
**Staphylococcus-sp.->Pseudoalteromonadaceae**	mutualExclusion	correl_pearson	-0.63576

**Enterococcus->Leptobacillium-leptobactrum**	mutualExclusion	dist_kullbackleibler	16.29102
**Streptococcus->Leuconostocaceae**	copresence	dist_kullbackleibler	0.048367
**Streptococcus->Lactococcus**	copresence	dist_kullbackleibler	0.004019
**Leuconostocaceae->Lactobacillus**	copresence	dist_kullbackleibler	0.021928
**Aspergillus-westerdijkiae->Alternaria**	mutualExclusion	dist_bray	0.986626
**Malassezia-restricta->Leptobacillium-leptobactrum**	copresence	correl_pearson	0.99518
**Mannheimia->Cobetia**	copresence	correl_spearman	0.804196
**Enhydrobacter->Mannheimia**	copresence	dist_kullbackleibler	0.05462
**Lactobacillaceae->Lactobacillus-delbrueckii**	copresence	dist_kullbackleibler	0.005599
**Streptococcus->Lactococcus**	copresence	dist_bray	0.023124
**Lactobacillaceae->Lactococcus**	copresence	correl_pearson	0.960198
**Acinetobacter-johnsonii->Streptococcus-equi**	mutualExclusion	correl_spearman	-0.62499
**Alternaria-alternata->Lactobacillus**	mutualExclusion	correl_pearson	-0.50738
**Lactobacillus-delbrueckii->Leuconostocaceae**	copresence	dist_bray	0.084206

**Psychrobacter->Meyerozyma-guilliermondii**	mutualExclusion	correl_pearson	-0.56468
**Aspergillus->Geosmithia-sp.**	mutualExclusion	dist_kullbackleibler	19.76448
**Salinivibrio-costicola->Cobetia**	copresence	correl_spearman	0.888112
**Pseudoalteromonadaceae->Lactobacillus-sp**	mutualExclusion	correl_spearman	-0.77728
**Salinivibrio-costicola->Staphylococcus-sp.**	copresence	correl_spearman	0.832168
**Candida-apicola->Leuconostoc**	copresence	correl_spearman	0.841132
**Debaryomyces-prosopidis->Pseudoalteromonadaceae**	copresence	correl_pearson	0.872044
**Streptococcus->Stemphylium**	copresence	correl_pearson	0.945705
**Staphylococcus->Lactobacillaceae**	mutualExclusion	correl_spearman	-0.62384
**Lactococcus->Lactobacillus**	copresence	dist_kullbackleibler	0.024837
**Alternaria->Botryosphaeria-dothidea**	mutualExclusion	dist_kullbackleibler	16.44953
**Lactococcus->Leuconostocaceae**	copresence	dist_bray	0.081851
**Saccharomyces-cerevisiae->Streptococcus-equi**	mutualExclusion	correl_pearson	-0.50572
**Lactococcus->Lactobacillus-delbrueckii**	copresence	dist_bray	0.019801

**Lactobacillus-delbrueckii->Lactobacillus**	copresence	dist_bray	0.065587
**Verticillium-leptobactrum->Malassezia-restricta**	copresence	correl_spearman	0.877388
**Geosmithia-sp.->Candida-magnoliae**	mutualExclusion	dist_kullbackleibler	21.60692
**Quambalaria-cyanescens->Verticillium-leptobactrum**	copresence	correl_pearson	0.892001
**Aspergillus-westerdijkiae->Alternaria**	mutualExclusion	dist_kullbackleibler	19.35066
**Meyerozyma-guilliermondii->Lactobacillus-sp**	mutualExclusion	correl_spearman	-0.63398
**Lactobacillaceae->Leuconostocaceae**	copresence	dist_kullbackleibler	0.023542
**Lactobacillus->Streptococcus**	copresence	dist_kullbackleibler	0.037327
**Sphingobacterium-faecium->Meyerozyma-guilliermondii**	mutualExclusion	correl_spearman	-0.6345
**Acinetobacter-johnsonii->Candida-apicola**	mutualExclusion	dist_bray	0.988052
**Salinivibrio-costicola->Staphylococcus-sp.**	copresence	dist_kullbackleibler	0.029291
**Candida-magnoliae->Enhydrobacter**	mutualExclusion	correl_spearman	-0.81213
**Hanseniaspora-nectarophila->Staphylococcus**	mutualExclusion	correl_spearman	-0.61123
**Lactobacillales->Lactobacillaceae**	copresence	correl_pearson	0.868297

**Wickerhamomyces-anomalus->Botryosphaeria-dothidea**	mutualExclusion	dist_kullbackleibler	18.61665
**Wickerhamomyces-anomalus->Corynebacterium-sp**	mutualExclusion	dist_bray	0.985651
**Streptococcus-equi->Candida-diddensiae**	mutualExclusion	correl_pearson	-0.52419
**Bacteroidales->Wickerhamomyces-anomalus**	mutualExclusion	correl_spearman	-0.69016
**Candida-diddensiae->Enhydrobacter**	mutualExclusion	correl_pearson	-0.50534
**Staphylococcus-sp.->Leuconostocaceae**	copresence	dist_bray	0.07715
**Stemphylium->Alternaria**	copresence	correl_spearman	0.842606
**Acinetobacter-johnsonii->Ralstonia**	mutualExclusion	correl_pearson	-0.56003
**Lactobacillus-delbrueckii->Stemphylium**	copresence	correl_pearson	0.958527
**Ralstonia->Vibrio-rumoiensis**	copresence	correl_spearman	0.851422
**Streptococcus-sp->Porphyromonadaceae**	mutualExclusion	correl_spearman	-0.62703
**Hanseniaspora-guilliermondii->Cladosporium-sphaerospermum**	copresence	correl_pearson	0.903367
**Staphylococcus-sciuri->Staphylococcus-sp.**	copresence	correl_spearman	0.82663
**Bacteroidales->Marinilactibacillus-psychrotolerans**	mutualExclusion	correl_pearson	-0.57379
**Acinetobacter-johnsonii->Lactobacillus-delbrueckii**	mutualExclusion	correl_spearman	-0.60464

**Botryosphaeria-dothidea->Candida-apicola**	mutualExclusion	dist_kullbackleibler	17.63755
**Lactobacillaceae->Streptococcus**	copresence	correl_pearson	0.932438
**Streptococcus-sp->Porphyromonadaceae**	mutualExclusion	correl_pearson	-0.69009
**Lactococcus->Stemphylium**	copresence	correl_pearson	0.929535
**Alternaria->Acinetobacter-johnsonii**	mutualExclusion	correl_pearson	-0.53766
